# Ultra-Processed Food Consumption Is Independently Associated with Higher HbA1c and Poor Glycemic Control in Adults with Type 2 Diabetes

**DOI:** 10.3390/nu18121951

**Published:** 2026-06-17

**Authors:** Nezihe Otay Lule, Serpil Şahin, Kemal Ozan Lule, Hamit Yildiz

**Affiliations:** 1 Department of Nutrition and Dietetics, Faculty of Health Sciences, Gaziantep University, 27310 Gaziantep, Türkiye; 2 Department of Internal Medicine, Faculty of Medicine, Gaziantep University, 27310 Gaziantep, Türkiye; serpilsahin.6327@gmail.com (S.Ş.); drkemalozanlule@gmail.com (K.O.L.); drhyildiz@hotmail.com (H.Y.)

**Keywords:** ultra-processed food, type 2 diabetes mellitus, HbA1c, glycemic control, sQ-HPF, cardiometabolic risk

## Abstract

**Background/Objectives:** Ultra-processed food (UPF) consumption has been linked to type 2 diabetes mellitus (T2DM) incidence, but its association with glycemic control in individuals already living with T2DM remains less well characterized. We evaluated the relationship between UPF consumption, assessed with the Turkish version of the short Screening Questionnaire of Highly Processed Food Consumption (sQ-HPF), and HbA1c and poor glycemic control in adults with T2DM. **Methods:** In this single-centre, cross-sectional observational study, 425 adults aged 18–65 years with T2DM of at least 6 months’ duration were consecutively recruited at a tertiary general internal medicine outpatient clinic. UPF consumption was assessed with the Turkish sQ-HPF (range 0–11; scores ≥ 6 = high UPF consumption). HbA1c was the primary outcome (continuous and dichotomized at 7.0%). Multiple linear and binary logistic regression analyses were performed with hierarchical adjustment for age, sex, body mass index (BMI), diabetes duration, and insulin-based treatment. **Results:** Total UPF score was positively correlated with HbA1c (r = 0.423, *p* < 0.001). In the fully adjusted linear model, each one-point increase in total UPF score was associated with a 0.418% higher HbA1c (95% CI: 0.333–0.503; β = 0.413; *p* < 0.001), explaining 28.9% of HbA1c variance. In the fully adjusted logistic model, each one-point increase in total UPF score was associated with 25.0% higher odds of poor glycemic control (OR = 1.250, 95% CI: 1.110–1.408, *p* < 0.001). Insulin-based treatment and male sex were also independent predictors. UPF score was not significantly associated with lipid parameters or C-reactive protein. **Conclusions:** Higher UPF consumption, captured by a brief validated screener, was independently associated with poorer glycemic control in Turkish adults with T2DM. Brief UPF screening may help identify patients who would benefit from targeted nutritional intervention.

## 1. Introduction

Type 2 diabetes mellitus (T2DM) remains one of the most important global public health challenges because of its increasing prevalence and its close relationship with cardiovascular, renal, metabolic, and functional complications. According to the International Diabetes Federation Diabetes Atlas, approximately 589 million adults aged 20–79 years are currently living with diabetes worldwide, and this number is projected to rise to approximately 853 million by 2050 [1]. This growing burden makes the identification of modifiable dietary factors associated with glycemic control and cardiometabolic risk particularly important in individuals already diagnosed with T2DM.

Ultra-processed foods (UPFs) have increasingly attracted attention in nutritional epidemiology. These foods are generally characterized by extensive industrial processing, high palatability, long shelf life, frequent use of additives, and relatively poor nutritional quality. According to the NOVA food classification system, ultra-processed foods correspond to NOVA Group 4 and are defined as industrial formulations made predominantly or entirely from substances derived from foods and additives, containing little if any intact whole food, and typically produced using processes and ingredients not used in domestic kitchens [2]. Representative examples include sugar-sweetened and artificially sweetened beverages, packaged snacks and confectionery, processed meats such as sausages and salami, instant and ready-to-eat products, industrial breads and breakfast cereals, and reconstituted or pre-prepared meals. Higher UPF consumption has been associated with increased risk of several non-communicable diseases, including T2DM. In three large prospective U.S. cohorts, higher total UPF intake was associated with greater T2DM risk, and the accompanying meta-analysis supported a positive association between UPF consumption and incident T2DM [3]. Similarly, a recent systematic review and updated meta-analysis reported that higher UPF consumption was associated with increased risk of diabetes, although the certainty of evidence and heterogeneity across studies should be considered [4].

Despite this growing evidence, much of the literature has focused on the role of UPF consumption in the development of T2DM rather than its potential association with glycemic control among individuals who already have the disease. This distinction is important because dietary exposures may have different implications for incident diabetes risk and for metabolic control in patients receiving diabetes care. Recent studies in adults with T2DM have begun to address this gap, but findings remain limited and somewhat inconsistent. Hudson et al. reported that the degree of food processing was associated with HbA1c levels in African American adults with T2DM, whereas Bauer et al. found no significant linear association between UPF consumption and glycemic markers but reported associations with total cholesterol and LDL-cholesterol [5,6]. These findings suggest that UPF consumption may be differentially related to glycemic and cardiometabolic parameters across populations, dietary patterns, and assessment methods.

Another methodological issue is the assessment of UPF consumption in clinical settings. Many previous studies have relied on detailed food records, 24 h dietary recalls, food-frequency questionnaires, or NOVA-based dietary classification. Although these approaches are valuable, they can be time-consuming and may be difficult to implement in routine clinical practice. The short Screening Questionnaire of Highly Processed Food Consumption (sQ-HPF) was developed as a practical tool to rapidly assess highly processed food consumption [7]. Its Turkish version has been validated and shown to be a reliable tool for assessing highly processed food consumption patterns in epidemiological and clinical research [8]. Rather than quantifying absolute portion sizes or energy intake, the sQ-HPF ranks individuals according to the habitual frequency of consumption across 11 ultra-processed food categories. Its ability to reflect actual UPF exposure has been formally assessed against reference methods: the Turkish version was validated against 3-day dietary records and showed a significant positive correlation with the percentage of energy derived from UPF, together with good test–retest reliability [8]. Accordingly, although dietary recalls and food-frequency questionnaires provide finer quantitative granularity, the sQ-HPF offers a validated and reproducible ranking of UPF exposure that is better suited to routine clinical use; its score should therefore be interpreted as a relative indicator of UPF intake rather than as an absolute measure of consumed quantity.

Evidence on the relationship between UPF consumption, glycemic control, and cardiometabolic risk in Turkish adults with T2DM remains scarce. Given the specific dietary culture, food environment, and clinical characteristics of this population, local data are needed. Therefore, the present study aimed to evaluate the association of UPF consumption, assessed using the Turkish version of the sQ-HPF, with glycemic control and cardiometabolic risk parameters in adults with T2DM. The primary objective was to examine the relationship between total UPF score and HbA1c. Secondary objectives were to compare glycemic, biochemical, anthropometric, and lifestyle parameters according to glycemic control and UPF consumption categories, and to determine whether total UPF score was independently associated with HbA1c and poor glycemic control after adjustment for relevant clinical covariates.

## 2. Materials and Methods

### 2.1. Study Design and Setting

This observational, analytical, cross-sectional study was conducted at the General Internal Medicine Outpatient Clinic of Gaziantep University Şahinbey Research and Practice Hospital, between 26 March 2026 and 22 May 2026. The study was designed to evaluate the relationship between UPF consumption and glycemic control and cardiometabolic risk parameters in adults with T2DM. The study population consisted of consecutively recruited adult patients with T2DM who attended the outpatient clinic during the study period and met the eligibility criteria. The study protocol specified that no additional laboratory tests or invasive procedures would be requested solely for research purposes; biochemical data were obtained from routine clinical assessments performed during outpatient care. The study protocol was approved by the Gaziantep University Non-Interventional Clinical Research Ethics Committee (protocol code 2026/220; date of approval 25 March 2026), and the study was conducted in accordance with the principles of the Declaration of Helsinki. Written informed consent was obtained from all participants prior to enrollment. The study was reported in accordance with the STROBE guidelines for cross-sectional studies.

### 2.2. Participants

Patients aged 18–65 years with a diagnosis of T2DM for at least 6 months were eligible for inclusion. Additional inclusion criteria were having routine blood tests available on the day of outpatient evaluation, being able to complete the questionnaire reliably, and providing informed consent.

Patients were excluded if they had a T2DM diagnosis for less than 6 months; were younger than 18 years or older than 65 years; had type 1 diabetes mellitus, maturity-onset diabetes of the young, or secondary diabetes; had undergone a change in diabetes medication or initiation of a new diabetes drug within the previous 3 months; had active malignancy or had received chemotherapy or radiotherapy within the previous year; had inflammatory bowel disease, celiac disease, or malabsorption syndrome; had undergone bariatric or gastrointestinal surgery within the previous 6 months; were pregnant or lactating; had chronic kidney disease stage 4–5 or were receiving renal replacement therapy; had active liver disease; had untreated or uncontrolled thyroid disease; had been hospitalized for diabetic ketoacidosis or hyperosmolar hyperglycemic state within the previous 3 months; had received high-dose systemic corticosteroid therapy for at least 1 month; were receiving enteral or parenteral nutrition or a physician-prescribed restrictive medical diet; or had cognitive impairment, psychiatric disease, or communication difficulties that prevented reliable questionnaire completion.

Participants were enrolled by consecutive sampling to minimize selection bias. During the study period, 634 consecutive adults with T2DM attending the outpatient clinic were assessed for eligibility. Of these, 186 did not meet the inclusion criteria or met one or more exclusion criteria, and 23 declined to participate or provided incomplete questionnaires; the final analytic sample therefore comprised 425 participants. The eligibility criteria were intended to yield a clinically homogeneous T2DM population with stable antidiabetic treatment, free of conditions that could independently affect glycemic, lipid, or inflammatory parameters, so that the association between UPF consumption and glycemic control could be assessed with minimal confounding.

### 2.3. Data Collection

Data were collected using a structured questionnaire prepared by the researchers and from routine clinical and laboratory records obtained during the outpatient visit. The questionnaire included sociodemographic characteristics, health-related variables, lifestyle characteristics, and selected dietary habits. Sociodemographic variables included age, sex, marital status, educational level, and income status.

Health-related variables included diabetes duration, diabetes treatment type, current antidiabetic medication classes, hypoglycemia within the last 3 months, physician-diagnosed chronic diseases, including hypertension and dyslipidemia, smoking status, and alcohol use. Antidiabetic medication classes were recorded separately as metformin, sulfonylureas, dipeptidyl peptidase-4 inhibitors, thiazolidinediones, sodium–glucose cotransporter-2 inhibitors, glucagon-like peptide-1 receptor agonists, and insulin therapy.

Coexisting physician-diagnosed conditions (hypertension and dyslipidemia) and all concurrent antidiabetic medication classes were systematically recorded so that the potential influence of comorbid conditions and simultaneous pharmacological treatments on glycemic control could be accounted for in the statistical models. In addition, patients with conditions or therapies likely to independently affect glycemic, lipid, or inflammatory parameters—including recent antidiabetic medication changes, active malignancy or recent chemotherapy/radiotherapy, advanced chronic kidney disease, active liver disease, uncontrolled thyroid disease, and recent high-dose systemic corticosteroid use—were excluded a priori, thereby minimizing confounding arising from coexisting pathologies or concurrent pharmacotherapy.

Lifestyle variables included regular physical activity during the previous 3 months and self-reported sleep quality. Dietary habit variables included the frequency of sugar-sweetened beverage consumption, fast-food consumption, and packaged snack consumption. Anthropometric measurements and biochemical parameters were also recorded and are described in detail below.

Diabetes treatment was recorded as oral antidiabetic drugs, insulin, or oral antidiabetic drugs plus insulin. For regression analyses, treatment was additionally dichotomized as oral antidiabetic drugs only versus insulin-based treatment, where insulin-based treatment included insulin alone or insulin combined with oral antidiabetic drugs.

### 2.4. Assessment of Ultra-Processed Food Consumption

UPF consumption was assessed using the Turkish version of the short Screening Questionnaire of Highly Processed Food Consumption (sQ-HPF). The sQ-HPF is an internationally developed and validated instrument: the original questionnaire was constructed and validated as a brief tool for evaluating highly processed food consumption within a large multicentre European study population [7], and the Turkish version used in the present study was subsequently validated and shown to be reliable for assessing highly processed food consumption in Turkish adults [8]. The full list of items and the scoring procedure are described below and are additionally provided in Supplementary Table S1. The questionnaire consists of 11 yes/no items covering different highly processed food groups, including full-fat dairy products, processed meats, fats, sugar-sweetened or artificially sweetened beverages, sweets, packaged snacks, ready-to-eat products, processed grains, sauces, discretionary added sugar or salt, and fried foods. Each “yes” response was scored as 1 point and each “no” response as 0 points. The total UPF score therefore ranged from 0 to 11, with higher scores indicating higher UPF consumption. In addition to using the total UPF score as a continuous variable, participants were categorized as having low or high UPF consumption according to the predefined cut-off used for the scale. In the present analysis, scores below 6 were classified as low UPF consumption and scores of 6 or higher were classified as high UPF consumption [8].

### 2.5. Anthropometric Measurements

Anthropometric measurements were obtained according to standard protocols. Body weight was measured using a calibrated digital scale with 0.1 kg precision. Height was measured using a stadiometer while participants stood in the Frankfort horizontal plane. Body mass index (BMI) was calculated as body weight in kilograms divided by height in meters squared. BMI categories were defined according to World Health Organization criteria [9].

Waist circumference was measured at the midpoint between the lowest rib and the iliac crest. Waist circumference risk categories were defined using sex-specific cut-off points. For men, waist circumference ≥94 cm was considered “risk” and ≥102 cm “high risk”; for women, waist circumference ≥80 cm was considered “risk” and ≥88 cm “high risk” [10].

### 2.6. Biochemical Measurements

Biochemical parameters were obtained from routine outpatient laboratory tests performed as part of standard clinical care. No additional blood sampling was performed for the study. All analyses were performed in the central laboratory of Gaziantep University Şahinbey Research and Practice Hospital, which participates in an external quality assessment programme and applies daily internal quality-control procedures in accordance with the manufacturers’ specifications. Venous blood samples were drawn after an overnight fast and analyzed on the day of collection. Fasting plasma glucose, triglycerides, total cholesterol, HDL-cholesterol, LDL-cholesterol, alanine aminotransferase, aspartate aminotransferase, urea, creatinine, and C-reactive protein were measured using a Beckman Coulter AU5800 clinical chemistry analyzer (Beckman Coulter, Inc., Brea, CA, USA) with the manufacturer’s original reagents. HbA1c was measured using a Lifotronic H9 glycated hemoglobin analyzer (Lifotronic Technology Co., Ltd., Shenzhen, China). The recorded biochemical variables included fasting plasma glucose, HbA1c, triglycerides, HDL-cholesterol, LDL-cholesterol, total cholesterol, alanine aminotransferase, aspartate aminotransferase, urea, creatinine, and C-reactive protein.

The primary outcome was HbA1c as a continuous variable. For categorical analyses, glycemic control status was classified according to the American Diabetes Association Standards of Care in Diabetes—2026, which recommend an HbA1c target of <7.0% (<53 mmol/mol) for many nonpregnant adults with diabetes. Accordingly, HbA1c values < 7.0% were categorized as adequate glycemic control, whereas HbA1c values ≥ 7.0% were categorized as suboptimal glycemic control [11].

### 2.7. Sample Size

The sample size was planned based on the effect size derived from the multivariable linear regression findings reported by Hudson et al. in adults with T2DM [5]. The a priori sample size was calculated using G*Power software (version 3.1.9.7; Heinrich-Heine-Universität Düsseldorf, Düsseldorf, Germany). Assuming an estimated small effect size of approximately Cohen’s f^2^ = 0.02, α = 0.05, 80% power, and a multivariable linear regression model including approximately six predictors, the minimum required sample size was estimated to be 395 participants. Considering possible missing data and measurement error, the target sample size was planned as approximately 410–420 participants. The final analysis included 425 participants.

### 2.8. Statistical Analyses

All statistical analyses were performed using IBM SPSS Statistics, version 24.0 (IBM Corporation, Armonk, NY, USA). The normality of continuous variables was assessed using the Shapiro–Wilk test, histograms, and Q–Q plots. No missing data were present for the variables included in the analyses, as all clinical, anthropometric, biochemical, and questionnaire data were complete for the 425 participants included; therefore, no imputation was required. Normally distributed continuous variables were presented as mean ± standard deviation, whereas non-normally distributed variables were presented as median and interquartile range. Categorical variables were summarized as frequencies and percentages. Participants were compared according to glycemic control status, defined as HbA1c < 7.0% and HbA1c ≥ 7.0%, and according to UPF consumption category, defined as low and high UPF consumption. For comparisons between two independent groups, normally distributed continuous variables were analyzed using the independent-samples *t*-test. Because triglycerides, ALT, and AST were not normally distributed, these variables were compared using the Mann–Whitney U test. Categorical variables were compared using Pearson’s chi-square test. Fisher’s exact test was used when expected cell counts were sparse.

Correlation analyses were performed to examine the associations between total UPF score and clinical, glycemic, biochemical, and cardiometabolic parameters. Pearson’s correlation coefficient was used for normally distributed variables, while Spearman’s rank correlation coefficient was used for triglycerides, ALT, and AST because these variables were non-normally distributed.

Multiple linear regression analysis was conducted to evaluate the association between total UPF score and HbA1c as a continuous dependent variable. Three hierarchical models were constructed. Model 1 was unadjusted and included only total UPF score. Model 2 was adjusted for age and sex. Model 3 was further adjusted for BMI, diabetes duration, and insulin-based treatment. Sex was coded as 0 = female and 1 = male. Insulin-based treatment was coded as 0 = oral antidiabetic drugs only and 1 = insulin or insulin plus oral antidiabetic drugs. Regression results were reported as unstandardized coefficients (B), standardized coefficients (β), 95% confidence intervals, and *p*-values. Multicollinearity was assessed using tolerance and variance inflation factor values.

In addition, binary logistic regression analysis was performed using poor glycemic control as the dependent variable, defined as HbA1c ≥ 7.0%. The same hierarchical modeling strategy was applied: Model 1 included total UPF score only, Model 2 was adjusted for age and sex, and Model 3 was additionally adjusted for BMI, diabetes duration, and insulin-based treatment. Logistic regression results were reported as odds ratios (ORs) with 95% confidence intervals. Model fit was evaluated using Nagelkerke R^2^ and the Hosmer–Lemeshow goodness-of-fit test.

To assess the robustness of the UPF–HbA1c association to potential residual confounding by pharmacologic treatment and unmeasured cardiometabolic factors, two prespecified sensitivity analyses were conducted in which the fully adjusted linear and logistic regression models were further expanded to include (i) each non-insulin antidiabetic drug class (metformin, sulfonylureas, dipeptidyl peptidase-4 inhibitors, thiazolidinediones, sodium–glucose cotransporter-2 inhibitors, and glucagon-like peptide-1 receptor agonists; each coded as use vs. non-use), and (ii) the same medication panel plus smoking history (ever vs. never), regular physical activity (yes vs. no), hypertension, and dyslipidemia. Stability of the UPF coefficient across these nested models was used as the criterion for robustness.

A two-sided *p*-value of <0.05 was considered statistically significant.

## 3. Results

### 3.1. General Characteristics According to Glycemic Control Status

General characteristics of participants according to glycemic control status are presented in Table 1. A total of 425 patients with type 2 diabetes mellitus were included in the analysis. Of these, 107 participants had HbA1c < 7.0%, while 318 participants had HbA1c ≥ 7.0%. Age did not differ significantly between groups. However, sex distribution was significantly different, with a higher proportion of men in the HbA1c ≥ 7.0% group compared with the HbA1c < 7.0% group. Overall, the sample was balanced by sex (219 women, 51.5%; 206 men, 48.5%); the significant difference therefore reflected the distribution of sex across glycemic control categories rather than an imbalance in the recruited sample as a whole. Participants with HbA1c ≥ 7.0% had a significantly longer diabetes duration and a higher total UPF score than those with HbA1c < 7.0%. The proportion of participants classified as having high UPF consumption was also significantly greater in the HbA1c ≥ 7.0% group. As expected, fasting glucose and HbA1c levels were markedly higher among participants with HbA1c ≥ 7.0%. Income status and diabetes treatment type differed significantly between glycemic control groups. Use of oral antidiabetic drugs alone was more frequent among participants with HbA1c < 7.0%, whereas insulin use and combined oral antidiabetic plus insulin therapy were more frequent among those with HbA1c ≥ 7.0%. Regarding cardiometabolic characteristics, BMI category, hypertension, dyslipidemia, and TG levels differed significantly between groups. TG levels were higher in the HbA1c ≥ 7.0% group, while HDL-C, LDL-C, total cholesterol, urea, creatinine, CRP, ALT, and AST did not differ significantly. Lifestyle variables including smoking status, alcohol use, regular exercise, sleep quality, sugar-sweetened beverage consumption, fast-food consumption, and packaged snack consumption were not significantly associated with glycemic control status.

### 3.2. Comparison According to UPF Consumption Categories

Table 2 presents the comparison of glycemic, cardiometabolic, and anthropometric parameters between participants with low and high UPF consumption. Additional sociodemographic, treatment-related, and lifestyle characteristics are provided in Supplementary Table S2. Participants in the high UPF group were significantly younger than those in the low UPF group. Glucose and HbA1c levels were significantly higher in the high UPF group, whereas BMI as a continuous variable did not differ significantly between groups. However, BMI category differed significantly according to UPF consumption status, with morbid obesity being more frequent among participants with high UPF consumption. No significant differences were observed between UPF groups in HDL-C, LDL-C, total cholesterol, TG, ALT, AST, urea, creatinine, CRP, or waist circumference risk category.

### 3.3. Correlations Between Total UPF Score and Clinical Parameters

Correlations between total UPF score and clinical, glycemic, biochemical, and cardiometabolic parameters are presented in Table 3. Total UPF score was positively correlated with glucose (r = 0.192, *p* < 0.001) and HbA1c (r = 0.423, *p* < 0.001), indicating that higher UPF consumption was associated with poorer glycemic parameters. The strongest correlation was observed between total UPF score and HbA1c. Total UPF score was negatively correlated with age (r = −0.175, *p* < 0.001) and diabetes duration (r = −0.128, *p* = 0.008), suggesting that higher UPF consumption tended to be observed among younger participants and those with shorter diabetes duration. However, total UPF score was not significantly correlated with BMI, HDL-C, LDL-C, total cholesterol, urea, creatinine, CRP, TG, ALT, or AST.

### 3.4. Multiple Linear Regression Analysis for HbA1c

Multiple linear regression analysis was performed to examine the association between total UPF score and HbA1c as a continuous outcome (Table 4). In the unadjusted model, total UPF score was positively associated with HbA1c (B = 0.429, 95% CI: 0.341 to 0.516, β = 0.423, *p* < 0.001). This association remained significant after adjustment for age and sex (B = 0.414, 95% CI: 0.325 to 0.503, β = 0.409, *p* < 0.001). In the fully adjusted model, total UPF score remained independently associated with HbA1c after adjustment for age, sex, BMI, diabetes duration, and insulin-based treatment (B = 0.418, 95% CI: 0.333 to 0.503, β = 0.413, *p* < 0.001). This indicates that each one-point increase in total UPF score was associated with a 0.418% higher HbA1c level. Male sex and insulin-based treatment were also independently associated with higher HbA1c levels. The fully adjusted model explained 28.9% of the variance in HbA1c.

### 3.5. Binary Logistic Regression for Poor Glycemic Control

Additional binary logistic regression analysis was performed using poor glycemic control, defined as HbA1c ≥ 7.0%, as the dependent variable (Table 5). In the unadjusted model, total UPF score was significantly associated with higher odds of poor glycemic control (OR = 1.208, 95% CI: 1.088–1.342, *p* < 0.001). This association remained significant after adjustment for age and sex (OR = 1.208, 95% CI: 1.084–1.346, *p* = 0.001). In the fully adjusted model, total UPF score remained independently associated with poor glycemic control after adjustment for age, sex, BMI, diabetes duration, and insulin-based treatment (OR = 1.250, 95% CI: 1.110–1.408, *p* < 0.001). Accordingly, each one-point increase in total UPF score was associated with 25.0% higher odds of having HbA1c ≥ 7.0%. Insulin-based treatment was also independently associated with poor glycemic control (OR = 3.688, 95% CI: 1.852–7.344, *p* < 0.001). BMI, diabetes duration, age, and sex were not independently associated with poor glycemic control in the fully adjusted model.

### 3.6. Sensitivity Analyses for Glycemic Control Models

Sensitivity analyses were performed to examine the robustness of the association between total UPF score and glycemic control after additional adjustment for medication- and lifestyle-related covariates. In the primary fully adjusted model, total UPF score remained independently associated with HbA1c as a continuous outcome (B = 0.418, 95% CI: 0.333–0.503; *p* < 0.001), and each 1-point increase in UPF score was associated with higher odds of poor glycemic control (OR = 1.250, 95% CI: 1.110–1.408; *p* < 0.001). After further adjustment for individual non-insulin antidiabetic drug classes, the association between UPF score and HbA1c remained virtually unchanged (β = 0.415, 95% CI: 0.333–0.497; *p* < 0.001). Similarly, UPF score remained significantly associated with poor glycemic control in logistic regression analysis (OR = 1.322, 95% CI: 1.154–1.514; *p* < 0.001). This model explained 36.6% of the variance in HbA1c and showed a Nagelkerke R^2^ of 0.368. In the second sensitivity model, which additionally included smoking history, regular physical activity, hypertension, and dyslipidemia, the association between UPF score and HbA1c also persisted (β = 0.408, 95% CI: 0.325–0.490; *p* < 0.001). The corresponding logistic regression model showed that each 1-point increase in UPF score was associated with 30.5% higher odds of poor glycemic control (OR = 1.305, 95% CI: 1.127–1.511; *p* < 0.001). This model explained 37.9% of HbA1c variance and had a Nagelkerke R^2^ of 0.448. Overall, these sensitivity analyses confirmed that the association between higher UPF consumption and poorer glycemic control was robust and was not materially attenuated after additional adjustment for antidiabetic medication classes, smoking history, physical activity, hypertension, and dyslipidemia.

### 3.7. Supplementary Findings According to UPF Consumption

In the supplementary analyses, dyslipidemia, sugar-sweetened beverage consumption, and packaged snack consumption differed significantly according to UPF consumption categories, whereas marital status, educational level, income status, diabetes treatment type, hypoglycemia history, hypertension, smoking status, alcohol use, regular exercise, sleep quality, and fast-food consumption did not differ significantly (Supplementary Table S2).

## 4. Discussion

In this cross-sectional study of 425 adults with type 2 diabetes mellitus (T2DM), higher ultra-processed food (UPF) consumption—assessed using the Turkish version of the short Screening Questionnaire of Highly Processed Food Consumption (sQ-HPF)—was independently associated with higher HbA1c and greater odds of poor glycemic control. Each one-point increase in total UPF score was associated with a 0.418% higher HbA1c in fully adjusted linear regression and a 25.0% higher odds of HbA1c ≥ 7.0% in binary logistic regression after adjustment for age, sex, body mass index (BMI), diabetes duration, and insulin-based treatment. These associations remained robust across hierarchical models, with total UPF score retaining the largest standardized effect on HbA1c (β = 0.413) alongside male sex and insulin-based treatment. Although the overall sample was balanced by sex, male sex was independently associated with higher HbA1c and was more frequent among patients with suboptimal glycemic control; this is consistent with previous observations of poorer glycemic target attainment in men with T2DM and was accounted for by including sex as a covariate in all adjusted models. The fully adjusted linear model explained 28.9% of HbA1c variance, indicating that, although substantial unexplained variability remains, UPF consumption is a quantitatively meaningful behavioral correlate of glycemic control in this population.

Our results converge with a rapidly growing body of evidence linking habitual UPF consumption to T2DM risk in the general population. In three large prospective U.S. cohorts, total UPF intake was associated with incident T2DM, with a positive linear trend across exposure categories [3]. An updated systematic review and meta-analysis of 14 cohort studies (*n* = 692,508) reported that the highest versus lowest UPF intake was associated with a 24% higher risk of diabetes, and each 10% increase in total UPF consumption corresponded to a 13% higher relative risk [4]. Comparable estimates were obtained in additional systematic reviews and umbrella reviews of prospective cohorts, which documented robust positive associations between UPF intake and incident T2DM, hypertension, and obesity [12,13,14]. The BMJ umbrella review by Lane et al. classified the evidence linking higher UPF intake to T2DM as “highly suggestive” (class II), placing diabetes among the most consistently supported metabolic outcomes of UPF exposure across observational research [12]. A 2025 state-of-the-evidence review further called for the explicit incorporation of UPF exposure into dietary guidelines for diabetes prevention [15]. Our study extends this incidence-focused literature by demonstrating that, even within an established T2DM population already receiving pharmacologic treatment, the cumulative dietary exposure captured by a brief UPF screener is independently associated with poorer glycemic control.

Evidence specifically targeting individuals with prevalent T2DM remains comparatively limited. Hudson et al., in 273 African American adults with T2DM, reported that the percentage of dietary grams from ultra-processed sources was positively associated with HbA1c (β_adj = 0.015; *p* = 0.032), with a 10% higher UPF share corresponding to approximately 0.28 percentage points higher HbA1c among non-insulin-treated patients [5]. Our absolute effect size—approximately 0.42 percentage points of HbA1c per one-point increment in the sQ-HPF total score—is broadly consistent with this gradient, although direct numerical comparison is limited by the different exposure scales (gram percentage vs. categorical screener). Conversely, the cross-sectional NUGLIC analysis by Bauer et al. did not find significant linear associations between quintiles of processed and ultra-processed food consumption and glycemic markers, but documented elevated total cholesterol and LDL-cholesterol in intermediate-to-high quintile categories compared with the lowest exposure group [6]. The heterogeneity between these studies likely reflects differences in population (ethnicity, baseline glycemic status, treatment regimens), dietary assessment instruments (24 h recalls, food-frequency questionnaires, or brief screeners), and statistical modeling. Our findings are also coherent with long-term prognostic data from the Moli-sani cohort, in which higher UPF consumption was associated with all-cause and cardiovascular mortality among 1065 participants with T2DM independently of overall diet quality [16], and with the UK Biobank prospective analysis in which UPF consumption increased the risk of diabetic microvascular complications, partly through inflammatory and lipid-related pathways [17]. Collectively, these observations suggest that UPF intake is a meaningful, modifiable behavioral correlate of metabolic deterioration along the full T2DM disease trajectory, from incidence to complications.

Several plausible biological pathways may underlie the observed association between UPF intake and HbA1c. UPFs are typically characterized by high refined-carbohydrate and added-sugar content, high glycemic load, low dietary fiber, and altered food matrices that favor faster digestion and steeper postprandial glucose excursions—features that, over time, are expected to elevate average glycemia [15,18]. Beyond macronutrient profile, food additives (including emulsifiers, non-nutritive sweeteners, and carrageenans) have been implicated in gut microbial dysbiosis, low-grade systemic inflammation, increased intestinal permeability, and impaired insulin sensitivity in mechanistic and large epidemiological studies [15,19]. UPFs are also hyperpalatable and energy-dense, which promotes passive overconsumption and adiposity—both of which adversely affect glycemic control [18,19]. Although the cross-sectional design of the present study precludes causal inference, the direction and magnitude of the observed associations are biologically plausible in light of these mechanisms. The association between UPF consumption and glycemic control was consistent across three complementary analytic approaches: a moderate positive correlation with HbA1c (r = 0.423), a continuous linear association in which each one-point increment in the sQ-HPF score corresponded to a 0.418% higher HbA1c, and a dichotomous logistic association in which each one-point increment was associated with 25.0% higher odds of poor glycemic control (HbA1c ≥ 7.0%). This convergence across correlational, continuous, and categorical models lends internal consistency to the finding. In the categorical analysis, UPF exposure was operationalized using the validated sQ-HPF cut-off (total score < 6, low; ≥6, high), while the continuous 0–11 score allowed assessment of dose; the progressive increase in HbA1c across the UPF score range is consistent with a dose-dependent relationship rather than a threshold effect. Notably, the UPF coefficient remained essentially unchanged after hierarchical adjustment for age, sex, BMI, diabetes duration, and insulin-based treatment, and was stable across both prespecified sensitivity analyses that additionally accounted for individual antidiabetic drug classes, smoking, physical activity, hypertension, and dyslipidemia. The stability of a graded, dose-dependent association despite progressively more conservative adjustment supports the interpretation that higher UPF consumption is independently associated with poorer glycemic control in adults with T2DM, in line with the title, although the cross-sectional design means this should be regarded as an association rather than a causal effect.

In contrast to some previous reports, we did not detect significant correlations between total UPF score and lipid parameters (HDL-C, LDL-C, total cholesterol, triglycerides) or C-reactive protein. Bauer et al., using quintiles of processed and ultra-processed food consumption, reported no significant association with glycemic parameters in patients with T2DM, although higher intake categories were associated with higher total and LDL-cholesterol [6]; in the general population, several cohort analyses have likewise linked UPF intake with adverse lipid profiles, systemic inflammation, and incident cardiovascular disease [20,21]. Several factors may explain the absence of these associations in our sample. First, a substantial proportion of patients with T2DM in routine outpatient care likely receive statin therapy as part of standard management, which would attenuate inter-individual variability in LDL-cholesterol and total cholesterol and bias associations toward the null; however, lipid-lowering treatment was not recorded in our database, so this hypothesis cannot be formally tested. Second, the sQ-HPF captures the frequency of consumption of broad food categories but does not quantify absolute energy or saturated fat intake, limiting its sensitivity to dose-dependent lipid effects. Third, the cross-sectional design and a single biochemical measurement cannot capture cumulative lipid burden. These methodological constraints should be considered before inferring that UPF intake has no lipid-related effect in patients with T2DM. A similar caution applies to the null association between UPF score and C-reactive protein (CRP). Although higher UPF intake has been cross-sectionally associated with greater high-sensitivity CRP concentrations in general-population cohorts, partly independent of body mass index [22], several factors may explain our null finding. First, established T2DM is itself accompanied by chronic low-grade systemic inflammation, which may raise baseline CRP across all participants and compress the inter-individual variability needed to detect a diet-related signal. Second, CRP was derived from a single routine measurement rather than serial or high-sensitivity assessment, increasing measurement variability and reducing sensitivity to modest dietary effects. Third, as a frequency-based screening instrument, the sQ-HPF does not quantify the absolute intake of additives or pro-inflammatory dietary components most plausibly linked to CRP. Accordingly, the present null finding should be interpreted as inconclusive rather than as evidence that UPF intake is unrelated to systemic inflammation in T2DM.

Although BMI as a continuous variable did not differ significantly between low- and high-UPF groups, the distribution across BMI categories differed substantially: morbid obesity (BMI ≥ 40 kg/m^2^) was almost three times more frequent in the high-UPF group (14.6% vs. 5.2%, *p* = 0.003). This pattern is consistent with prospective evidence linking higher UPF intake to weight gain and the risk of incident obesity, particularly at the upper end of the BMI spectrum [21,23]. The absence of a continuous BMI difference may reflect the high baseline prevalence of overweight and obesity in our T2DM sample (more than 82% of participants), which compresses inter-group variability, as well as the cross-sectional nature of the comparison. We therefore interpret the BMI-category finding as suggestive of clustering of high UPF intake with extreme adiposity, rather than as evidence of a graded dose–response relationship in this clinical sample.

A notable demographic finding was that high UPF consumers were younger than low UPF consumers (49.8 vs. 52.5 years; *p* = 0.012), and total UPF score was inversely correlated with both age and diabetes duration. This pattern aligns with international literature describing higher UPF intake among younger adults [19] and with recent Turkish data in which adults attending an internal medicine outpatient clinic in the highest tertile of NOVA-processed food intake had higher waist-to-hip ratios and Framingham risk scores than those in the lowest tertile [24]. The Turkish version of the sQ-HPF has been validated as a brief, reliable tool that captures clinically relevant variation in highly processed food consumption [8]. Taken together, these observations suggest that UPF-reduction strategies in T2DM care may need to be tailored to younger adults with shorter disease duration—a group sometimes perceived as having lower priority for intensive dietary counseling but who in fact carry higher exposure to UPF-related risk and a longer remaining horizon for cumulative metabolic damage.

The present study has several strengths. To our knowledge, this is one of the first investigations to evaluate the association between UPF consumption and glycemic control in Turkish adults with T2DM using a culturally adapted, validated, clinic-friendly screening instrument [8]. The relatively large sample size (*n* = 425), the use of hierarchical multivariable models, and the simultaneous reporting of continuous (HbA1c) and categorical (HbA1c ≥ 7.0%) outcomes increase the robustness of the findings. The exclusion of patients with recent medication changes, active malignancy, advanced kidney or liver disease, recent acute hyperglycemic events, and other major confounders enhances internal validity.

To probe the robustness of the UPF–HbA1c association to residual confounding by treatment intensity and cardiometabolic comorbidity, two prespecified sensitivity analyses were performed (Table 6). Sequential adjustment of the primary model for each non-insulin antidiabetic drug class (metformin, sulfonylureas, DPP-4 inhibitors, thiazolidinediones, SGLT2 inhibitors, and GLP-1 receptor agonists) yielded a UPF coefficient that was essentially unchanged from the primary estimate (β = 0.415, 95% CI 0.333–0.497, *p* < 0.001; model R^2^ = 0.366). Further adjustment for smoking history, regular physical activity, hypertension, and dyslipidemia produced near-identical estimates (β = 0.408, 95% CI 0.325–0.490, *p* < 0.001; model R^2^ = 0.379), corresponding to only a 1.6% relative attenuation of the UPF effect compared with the primary model. In the logistic models, the odds ratio for poor glycemic control per one-point increase in sQ-HPF score remained statistically significant and was modestly strengthened after additional adjustment for non-insulin antidiabetic drug classes (OR = 1.322, 95% CI 1.13–1.51). This pattern is consistent with negative (suppressor) confounding: because treatment intensity is reactive to glycemic control—patients with poorer control receive more intensive pharmacotherapy, which in turn lowers HbA1c—leaving medication unadjusted partially masks the underlying UPF–poor-control association, and adjusting for drug classes more fully reveals it. The magnitude of the change was small, the confidence intervals overlapped substantially with the primary estimate, and the direction and statistical significance were unchanged, indicating partial unmasking of a genuine association rather than estimate instability. The stability of the UPF estimate across these nested models argues against substantial residual confounding by antidiabetic regimen complexity or by major lifestyle and comorbid factors, and supports the interpretation that higher UPF consumption is independently associated with poorer glycemic control in this population.

Several limitations should be acknowledged. First, the cross-sectional design precludes causal inference; reverse causality—whereby patients with poorer glycemic control may have less stringent dietary self-regulation—cannot be excluded, and our findings should therefore be interpreted as associations rather than as evidence of causation. Second, the sQ-HPF is a brief screener and does not quantify portion sizes, total energy intake, or specific nutrients, limiting the granularity of exposure assessment compared with 24 h recalls or food-frequency questionnaires [5,6,7]. Third, self-report dietary instruments are subject to social desirability bias, which is expected to bias the observed associations toward the null. Fourth, the study was conducted at a single tertiary outpatient clinic. Patients attending a tertiary centre may have more advanced or longer-standing disease and/or better access to healthcare resources than individuals managed in community or primary-care settings, which may introduce selection bias and limit the representativeness of the sample. Consequently, caution is warranted when extrapolating these findings to the broader T2DM population, and multicentre studies enrolling participants from primary-care and community settings across different regions of Türkiye will be needed to confirm and generalize the present results. Fifth, although our sensitivity analyses accounted for the use of each major antidiabetic drug class, we did not capture antidiabetic drug dosage, treatment adherence, lipid-lowering or antihypertensive medication use, or objectively assessed physical activity, any of which could contribute to residual confounding. Sixth, although the fully adjusted model explained 28.9% of HbA1c variance, the majority of variation remains unexplained, reflecting the multifactorial nature of glycemic control.

From a clinical perspective, our findings suggest that brief, validated UPF screening tools such as the Turkish sQ-HPF can identify a modifiable dietary pattern associated with poorer glycemic control in routine T2DM care. Incorporating such instruments into outpatient practice may help clinicians prioritize patients—particularly younger adults with shorter diabetes duration but high UPF intake—who would benefit most from structured nutritional intervention. Future longitudinal studies and randomized controlled trials are needed to establish whether reducing UPF consumption translates into clinically meaningful improvements in HbA1c and downstream cardiometabolic outcomes in adults with T2DM.

## 5. Conclusions

In this cross-sectional study of Turkish adults with T2DM, higher UPF consumption, assessed using the validated Turkish sQ-HPF, was independently associated with higher HbA1c levels and greater odds of poor glycemic control after adjustment for age, sex, BMI, diabetes duration, and insulin-based treatment. UPF intake was not significantly associated with lipid parameters or CRP, although morbid obesity was more frequent among high UPF consumers. To our knowledge, this is one of the relatively few studies to quantify the independent association between UPF consumption and glycemic control specifically in adults with already-established T2DM using a validated screening instrument, whereas most prior evidence has been derived from general or non-diabetic populations. In our view, the consistency of this association across correlational, continuous, and categorical analyses, together with its stability under progressively more conservative adjustment, indicates that UPF consumption is a clinically relevant dietary correlate of glycemic control in this population rather than an incidental finding. These results are consistent with previous cohort and cross-sectional studies linking UPF intake to incident T2DM and adverse cardiometabolic profiles, and extend them by focusing on glycemic control within an already-diagnosed diabetic population. The brevity and feasibility of the sQ-HPF make it a promising tool for routine dietary screening in T2DM outpatient care, particularly in younger patients with shorter diabetes duration. Longitudinal and interventional studies are required to confirm causality and to evaluate whether targeted UPF reduction improves long-term metabolic outcomes.

## Figures and Tables

**Table 1:** General characteristics of participants according to glycemic control status.

Variable	Total (*n* = 425)	HbA1c < 7.0% (*n* = 107)	HbA1c ≥ 7.0% (*n* = 318)	*p*-Value
**Age, years**	51.29 ± 11.17	51.46 ± 11.24	51.24 ± 11.16	0.859
**Sex**				0.027 *
Female	219 (51.5)	65 (60.7)	154 (48.4)	
Male	206 (48.5)	42 (39.3)	164 (51.6)	
**Marital status**				0.968
Single	67 (15.8)	17 (15.9)	50 (15.7)	
Married	358 (84.2)	90 (84.1)	268 (84.3)	
**Educational level**				0.163
Illiterate/primary school	254 (59.8)	56 (52.3)	198 (62.3)	
Secondary/high school	112 (26.4)	35 (32.7)	77 (24.2)	
University or higher	59 (13.9)	16 (15.0)	43 (13.5)	
**Income status**				0.004 *
Income higher than expenses	28 (6.6)	8 (7.5)	20 (6.3)	
Income equal to expenses	190 (44.7)	33 (30.8)	157 (49.4)	
Income lower than expenses	207 (48.7)	66 (61.7)	141 (44.3)	
**BMI, kg/m^2^**	30.67 ± 6.22	31.77 ± 6.62	30.30 ± 6.05	0.044 *
**BMI category**				0.006 *
Underweight	2 (0.5)	0 (0.0)	2 (0.6)	
Normal weight	73 (17.2)	13 (12.1)	60 (18.9)	
Overweight	140 (32.9)	41 (38.3)	99 (31.1)	
Obese	170 (40.0)	35 (32.7)	135 (42.5)	
Morbidly obese	40 (9.4)	18 (16.8)	22 (6.9)	
**Diabetes duration, years**	8.07 ± 7.00	6.51 ± 5.72	8.60 ± 7.31	0.003 *
**Diabetes treatment type**				<0.001 *
Oral antidiabetic drugs	294 (69.2)	93 (86.9)	201 (63.2)	
Insulin	34 (8.0)	4 (3.7)	30 (9.4)	
Oral antidiabetic drugs + insulin	97 (22.8)	10 (9.3)	87 (27.4)	
**Hypoglycemia in the last 3 months**				0.194
No	352 (82.8)	93 (86.9)	259 (81.4)	
Yes	73 (17.2)	14 (13.1)	59 (18.6)	
**Hypertension**				<0.001 *
No	230 (54.1)	37 (34.6)	193 (60.7)	
Yes	195 (45.9)	70 (65.4)	125 (39.3)	
**Dyslipidemia**				0.018 *
No	164 (38.6)	31 (29.0)	133 (41.8)	
Yes	261 (61.4)	76 (71.0)	185 (58.2)	
Glucose, mg/dL	189.61 ± 86.93	125.57 ± 34.98	211.15 ± 88.59	<0.001 *
HbA1c, %	8.70 ± 2.32	6.21 ± 0.45	9.53 ± 1.95	<0.001 *
HDL-C, mg/dL	47.08 ± 13.04	48.08 ± 11.63	46.74 ± 13.48	0.358
LDL-C, mg/dL	126.68 ± 37.14	129.22 ± 35.70	125.82 ± 37.63	0.413
Total cholesterol, mg/dL	213.99 ± 48.96	212.82 ± 46.58	214.38 ± 49.80	0.776
TG, mg/dL	174.0 (122.0–249.5)	163.0 (106.0–215.00)	180.5 (124.0–262.5)	0.047 *
ALT, U/L	21 (16–30)	20 (17–30)	22 (16–29)	0.623
AST, U/L	18 (15–23)	18 (14–22)	18 (15–23)	0.745
Urea, mg/dL	31.75 ± 11.39	30.97 ± 11.36	32.02 ± 11.41	0.413
Creatinine, mg/dL	0.71 ± 0.20	0.72 ± 0.19	0.71 ± 0.20	0.814
CRP, mg/L	4.94 ± 3.45	4.61 ± 3.59	5.06 ± 3.40	0.241
WC risk category				0.336
No risk	48 (11.3)	8 (7.5)	40 (12.6)	
Risk	53 (12.5)	13 (12.1)	40 (12.6)	
High risk	324 (76.2)	86 (80.4)	238 (74.8)	
**Total UPF score**	5.24 ± 2.04	4.58 ± 2.14	5.46 ± 2.18	<0.001 *
**UPF category**				0.003 *
Low UPF	233 (54.8)	72 (67.3)	161 (50.6)	
High UPF	192 (45.2)	35 (32.7)	157 (49.4)	
**Smoking status**				0.867
Current smoker	107 (25.2)	29 (27.1)	78 (24.5)	
Non-smoker	293 (68.9)	72 (67.3)	221 (69.5)	
Former smoker	25 (5.9)	6 (5.6)	19 (6.0)	
**Alcohol use**				0.685
No	418 (98.4)	106 (99.1)	312 (98.1)	
Yes	7 (1.6)	1 (0.9)	6 (1.9)	
**Regular exercise**				0.465
No	302 (71.1)	79 (73.8)	223 (70.1)	
Yes	123 (28.9)	28 (26.2)	95 (29.9)	
**Sleep quality**				0.303
Good	126 (29.6)	27 (25.2)	99 (31.1)	
Moderate	204 (48.0)	51 (47.7)	153 (48.1)	
Poor	95 (22.4)	29 (27.1)	66 (20.8)	
**Sugar-sweetened beverage consumption**				0.325
Never/rarely	237 (55.8)	61 (57.0)	176 (55.3)	
1–3 times/month	101 (23.8)	25 (23.4)	76 (23.9)	
1–2 times/week	64 (15.1)	13 (12.1)	51 (16.0)	
>3 times/week	7 (1.6)	4 (3.7)	3 (0.9)	
Daily	16 (3.8)	4 (3.7)	12 (3.8)	
**Fast-food consumption**				0.333
Never/rarely	351 (82.6)	84 (78.5)	267 (84.0)	
1–3 times/month	38 (8.9)	13 (12.1)	25 (7.9)	
1–2 times/week	33 (7.8)	10 (9.3)	23 (7.2)	
>3 times/week	3 (0.7)	0 (0.0)	3 (0.9)	
**Packaged snack consumption**				0.320
Never/rarely	267 (62.8)	71 (66.4)	196 (61.6)	
1–3 times/month	102 (24.0)	20 (18.7)	82 (25.8)	
1–2 times/week	48 (11.3)	13 (12.1)	35 (11.0)	
>3 times/week	6 (1.4)	3 (2.8)	3 (0.9)	
Daily	2 (0.5)	0 (0.0)	2 (0.6)	

Data are presented as mean ± standard deviation, median (interquartile range), or *n* (%), as appropriate. Percentages for categorical variables are column percentages within HbA1c categories. Normally distributed continuous variables were compared using the independent-samples *t*-test. TG, ALT, and AST were compared using the Mann–Whitney U test. Categorical variables were compared using Pearson’s chi-square test, except where Fisher’s exact test was required because of sparse expected cell counts. * *p* < 0.05. BMI, body mass index; UPF, ultra-processed food; HDL-C, high-density lipoprotein cholesterol; LDL-C, low-density lipoprotein cholesterol; TG, triglycerides; ALT, alanine aminotransferase; AST, aspartate aminotransferase; CRP, C-reactive protein; WC, waist circumference.

**Table 2:** Comparison of glycemic, cardiometabolic, and anthropometric parameters according to UPF consumption categories.

Variable	Total (*n* = 425)	Low UPF (*n* = 233)	High UPF (*n* = 192)	*p*-Value
**Age, years**	51.29 ± 11.17	52.53 ± 10.93	49.79 ± 11.30	0.012 *
**Sex**				0.053
Female	219 (51.5)	130 (55.8)	89 (46.4)	
Male	206 (48.5)	103 (44.2)	103 (53.6)	
**BMI, kg/m^2^**	30.67 ± 6.22	30.26 ± 5.26	31.17 ± 7.20	0.145
**BMI category**				0.003 *
Underweight	2 (0.5)	0 (0.0)	2 (1.0)	
Normal weight	73 (17.2)	41 (17.6)	32 (16.7)	
Overweight	140 (32.9)	75 (32.2)	65 (33.9)	
Obese	170 (40.0)	105 (45.1)	65 (33.9)	
Morbidly obese	40 (9.4)	12 (5.2)	28 (14.6)	
**WC risk category**				0.960
No risk	48 (11.3)	26 (11.2)	22 (11.5)	
Risk	53 (12.5)	30 (12.9)	23 (12.0)	
High risk	324 (76.2)	177 (76.0)	147 (76.6)	
Glucose, mg/dL	189.61 ± 86.93	178.04 ± 81.02	203.65 ± 91.87	0.003 *
HbA1c, %	8.70 ± 2.23	7.98 ± 1.76	9.57 ± 2.43	<0.001 *
HDL-C, mg/dL	47.08 ± 13.04	46.95 ± 13.07	47.23 ± 13.03	0.825
LDL-C, mg/dL	126.68 ± 37.14	127.27 ± 38.84	125.96 ± 35.06	0.715
Total cholesterol, mg/dL	213.9 ± 48.9	213.1 ± 50.8	215.1 ± 46.7	0.667
TG, mg/dL	174.0 (122.0–249.5)	173.0 (121.0–237.5)	179.0 (122.3–264.0)	0.559
ALT, U/L	21 (16–30)	22 (16–31)	21 (16–28)	0.382
AST, U/L	18 (15–23)	18 (15–23)	18 (14–22)	0.235
Urea, mg/dL	31.75 ± 11.39	32.09 ± 11.62	31.35 ± 11.12	0.508
Creatinine, mg/dL	0.71 ± 0.20	0.72 ± 0.20	0.70 ± 0.20	0.291
CRP, mg/L	4.94 ± 3.45	4.83 ± 3.37	5.08 ± 3.56	0.465

Data are presented as mean ± standard deviation, median (interquartile range), or *n* (%), as appropriate. Percentages are column percentages within UPF consumption categories. Normally distributed continuous variables were compared using the independent-samples *t*-test. TG, ALT, and AST were compared using the Mann–Whitney U test. Categorical variables were compared using Pearson’s chi-square test. * *p* < 0.05. UPF, ultra-processed food; BMI, body mass index; HbA1c, glycated hemoglobin; HDL-C, high-density lipoprotein cholesterol; LDL-C, low-density lipoprotein cholesterol; TG, triglycerides; ALT, alanine aminotransferase; AST, aspartate aminotransferase; CRP, C-reactive protein; WC, waist circumference.

**Table 3:** Correlations between total UPF score and clinical, glycemic, biochemical, and cardiometabolic parameters (*n* = 425).

Variable	r/ρ	*p*-Value
Age	−0.175	<0.001 *
BMI	0.044	0.363
Diabetes duration	−0.128	0.008 *
Glucose	0.192	<0.001 *
HbA1c	0.423	<0.001 *
HDL-C	0.043	0.372
LDL-C	−0.004	0.941
Total cholesterol	0.034	0.482
Urea	−0.022	0.647
Creatinine	−0.040	0.416
CRP	0.015	0.751
TG	0.035	0.473
ALT	−0.006	0.895
AST	−0.035	0.468

Pearson’s correlation coefficient was used for normally distributed variables and is denoted by r. Spearman’s rank correlation coefficient was used for TG, ALT, and AST because these variables were not normally distributed and is denoted by ρ. * *p* < 0.05. BMI, body mass index; UPF, ultra-processed food; HDL-C, high-density lipoprotein cholesterol; LDL-C, low-density lipoprotein cholesterol; TG, triglycerides; ALT, alanine aminotransferase; AST, aspartate aminotransferase; CRP, C-reactive protein.

**Table 4:** Multiple linear regression analysis for HbA1c (*n* = 425).

Variable	Model 1 B (95% CI)	β	*p*-Value	Model 2 B (95% CI)	β	*p*-Value	Model 3 B (95% CI)	β	*p*-Value
Total UPF score	0.429 (0.341 to 0.516)	0.423	<0.001 *	0.414 (0.325 to 0.503)	0.409	<0.001 *	0.418 (0.333 to 0.503)	0.413	<0.001 *
Age, years	—	—	—	−0.003 (−0.021 to 0.014)	−0.017	0.707	0.012 (−0.006 to 0.030)	0.059	0.195
Male sex	—	—	—	0.541 (0.154 to 0.928)	0.121	0.006 *	0.623 (0.243 to 1.003)	0.140	0.001 *
BMI, kg/m^2^	—	—	—	—	—	—	−0.019 (−0.050 to 0.012)	−0.053	0.236
Diabetes duration, years	—	—	—	—	—	—	0.016 (−0.014 to 0.045)	0.049	0.300
Insulin-based treatment	—	—	—	—	—	—	1.361 (0.907 to 1.815)	0.282	<0.001 *
Model fit	Model 1: R^2^ = 0.179, adjusted R^2^ = 0.177, *p* < 0.001 *			Model 2: R^2^ = 0.194, adjusted R^2^ = 0.188, *p* < 0.001 *			Model 3: R^2^ = 0.289, adjusted R^2^ = 0.278, *p* < 0.001 *		

The dependent variable was HbA1c (%). Model 1 was unadjusted. Model 2 was adjusted for age and sex. Model 3 was adjusted for age, sex, BMI, diabetes duration, and insulin-based treatment. Sex was coded as 0 = female and 1 = male. Insulin-based treatment was coded as 0 = oral antidiabetic drugs only and 1 = insulin or insulin plus oral antidiabetic drugs. B represents the unstandardized regression coefficient; β represents the standardized regression coefficient. * *p* < 0.05. UPF, ultra-processed food; BMI, body mass index.

**Table 5:** Binary logistic regression analysis of independent predictors of poor glycemic control (*n* = 425).

Variable	Model 1 OR 95% CI	*p*-Value	Model 2 OR 95% CI	*p*-Value	Model 3 OR 95% CI	*p*-Value
Total UPF score	1.208 (1.088–1.342)	<0.001 *	1.208 (1.084–1.346)	0.001 *	1.250 (1.110–1.408)	<0.001 *
Age, years	—	—	1.007 (0.987–1.028)	0.495	1.021 (0.996–1.046)	0.097
Male sex	—	—	1.577 (1.000–2.486)	0.050	1.629 (0.999–2.656)	0.051
BMI, kg/m^2^	—	—	—	—	0.970 (0.933–1.010)	0.139
Diabetes duration, years	—	—	—	—	1.026 (0.983–1.070)	0.238
Insulin-based treatment	—	—	—	—	3.688 (1.852–7.344)	<0.001 *
Model fit	Model 1 Nagelkerke R^2^ = 0.045; Hosmer–Lemeshow *p* = 0.254		Model 2 Nagelkerke R^2^ = 0.059; Hosmer–Lemeshow *p* = 0.850		Model 3 Nagelkerke R^2^ = 0.159; Hosmer–Lemeshow *p* = 0.254	

The dependent variable was poor glycemic control, defined as HbA1c ≥ 7.0%. Model 1 was unadjusted. Model 2 was adjusted for age and sex. Model 3 was adjusted for age, sex, BMI, diabetes duration, and insulin-based treatment. Odds ratios are presented with 95% confidence intervals. Sex was coded as 0 = female and 1 = male. Insulin-based treatment was coded as 0 = oral antidiabetic drugs only and 1 = insulin or insulin plus oral antidiabetic drugs. * *p* < 0.05. UPF, ultra-processed food; BMI, body mass index.

**Table 6:** Sensitivity analyses for the association between sQ-HPF score and glycemic control in patients with type 2 diabetes (*n* = 425).

Model	Additional Covariates Beyond Primary Model	UPF B (95% CI)	*p*	R^2^	UPF OR (95% CI)	*p*	Nagelkerke R^2^
Primary (Model 3)	— (age, sex, BMI, diabetes duration, insulin-based treatment)	0.418 (0.333–0.503)	<0.001	0.289	1.250 (1.110–1.408)	<0.001	0.159
Sensitivity 1	+ each non-insulin antidiabetic drug class ª	0.415 (0.333–0.497)	<0.001	0.366	1.322 (1.154–1.514)	<0.001	0.368
Sensitivity 2	+ smoking history, regular physical activity, hypertension, dyslipidemia	0.408 (0.325–0.490)	<0.001	0.379	1.305 (1.127–1.511)	<0.001	0.448

The linear regression dependent variable was HbA1c (%); the logistic regression dependent variable was poor glycemic control (HbA1c ≥ 7.0%). All models include the covariates of the primary fully adjusted model (age, sex, BMI, diabetes duration, insulin-based treatment). In the “Additional Covariates Beyond Primary Model” column, “—” denotes that no covariates were entered beyond the primary model, whereas “+” indicates the additional covariates included in each sensitivity analysis. UPF intake was operationalized using the sQ-HPF total score; thus “UPF” in the column headers refers to this score. B represents the unstandardized regression coefficient for the sQ-HPF total score (per 1-point increment). The odds ratio (OR) likewise expresses the change in odds of poor glycemic control per 1-point increment in sQ-HPF score. ª Non-insulin antidiabetic drug classes entered as separate binary indicators (use vs. non-use): metformin, sulfonylureas, dipeptidyl peptidase-4 inhibitors (DPP-4i), thiazolidinediones (TZD), sodium–glucose cotransporter-2 inhibitors (SGLT2i), and glucagon-like peptide-1 receptor agonists (GLP-1 RA). CI, confidence interval; sQ-HPF, short Questionnaire on Highly Processed Food consumption; UPF, ultra-processed food; OR, odds ratio.

## Data Availability

The datasets generated and/or analyzed during the current study are not publicly available due to institutional data protection policies but are available from the corresponding author on reasonable request.

## Supplementary Materials

The following supporting information can be downloaded at: https://www.mdpi.com/article/10.3390/nu18121951/s1, Table S1: The 11-item Turkish version of the short Screening Questionnaire of Highly Processed Food Consumption (sQ-HPF); Table S2: Sociodemographic, clinical treatment, and lifestyle characteristics according to UPF consumption categories.

## Abbreviations

The following abbreviations are used in this manuscript:

ALT	Alanine aminotransferase
AST	Aspartate aminotransferase
BMI	Body mass index
CRP	C-reactive protein
HbA1c	Glycated hemoglobin
HDL-C	High-density lipoprotein cholesterol
LDL-C	Low-density lipoprotein cholesterol
OAD	Oral antidiabetic drug
sQ-HPF	Short Screening Questionnaire of Highly Processed Food Consumption
T2DM	Type 2 diabetes mellitus
TG	Triglycerides
UPF	Ultra-processed food
WC	Waist circumference
