# Supplementary material for: Ultra-Processed Food Consumption Is Independently Associated with Higher HbA1c and Poor Glycemic Control in Adults with Type 2 Diabetes
Source: Nutrients. 2026 Jun 17;18(12):1951. doi: 10.3390/nu18121951 (PMC13304910; doi:10.3390/nu18121951)
Supplement: Supplementary file 1 [file nutrients-18-01951-s001.zip › Supplementary Table S1 revised.pdf]

Supplementary Table S1. The 11-item Turkish version of the short Screening Questionnaire of Highly Processed Food Consumption (sQ-HPF)

No	Food group	Foods and quantities	Frequency threshold	Yes (1 point)	No (0 points)
1	Fatty dairy products	Cream (120 g), smoked or cured cheese (50 g), old cheddar (50 g), soft cheese (2 wedges, 25 g)	≥2 times/week	☐	☐
2	Cured meats	Ham (30 g), salami/sucuk/sausage/bacon (50 g), sandwich with salami/sucuk/sausage/bacon (30 g), cured cold meat (50 g), pâté (25 g)	≥1 time/day	☐	☐
3	Fats	Margarine (12 g), butter (12 g), lard/animal fat (10 g)	>3 times/month	☐	☐
4	Sugary and artificially sweetened drinks	Soft drinks (200 mL, 1 small can), artificially sweetened drinks (200 mL, 1 small can), bottled juices (200 mL, 1 small can), grape must/hardaliye (100 mL)	≥2 times/week	☐	☐
5	Sweets	Ice cream/sorbet (70 g), canned fruit (2 pieces), biscuits (50 g), whole-grain biscuits (50 g), chocolate biscuits (50 g), honey (1 dessert spoon), molasses (1 dessert spoon), oven-baked homemade desserts (50 g), candies (50 g), donut (1 piece), cakes (muffin, cupcake, etc.) (1–2 pieces/slice), packaged cakes (50 g), sherbet desserts (100 g), chocolates (30 g), chocolate/cocoa drink powders (1 dessert spoon), hazelnut spread/cream (40 g), marzipan/flour cookies (90 g), jam (1 dessert spoon)	>1 time/day	☐	☐
6	Snacks	Packaged potato crisps (50 g), packaged snacks (50 g)	>3 times/month	☐	☐
7	Ready-to-eat products	Pizza (1 portion, 200 g), croquettes (1 portion), instant soup (1 bowl/plate)	>3 times/month	☐	☐
8	Refined cereals	White bread (75 g, 3 slices), sliced packaged bread (75 g, 3 slices), breakfast cereals (30 g), spaghetti/pasta/noodles (dry weight 60 g), white rice (dry weight 60 g)	≥2 times/week	☐	☐
9	Sauces	Mustard (1 dessert spoon), mayonnaise (1 dessert spoon), tomato sauce/ketchup (1 dessert spoon)	>1 time/week	☐	☐
10	Additives	Sugar (1 dessert spoon), table salt (1 pinch)	>3 times/day	☐	☐
11	Fried foods	Eat-out or homemade fried foods	≥2 times/week	☐	☐

A "Yes" response (1 point) is recorded when the participant consumes the corresponding food group at or above the indicated frequency threshold; otherwise a "No" response (0 points) is recorded. The total sQ-HPF score is the sum of all "Yes" responses (range 0–11); higher scores indicate higher ultra-processed (highly processed) food consumption, and a score ≥6 denotes high consumption. The instrument, food examples, and reference quantities are reproduced from the validated Turkish version of the sQ-HPF (Erdoğan Gövez et al., 2024 [8]), culturally adapted from the original sQ-HPF (Martínez-Pérez et al., 2022 [7]). Reproduced under the terms of the Creative Commons Attribution (CC BY 4.0) license.
