# Supplementary material for: Ultra-Processed Food Consumption Is Independently Associated with Higher HbA1c and Poor Glycemic Control in Adults with Type 2 Diabetes
Source: Nutrients. 2026 Jun 17;18(12):1951. doi: 10.3390/nu18121951 (PMC13304910; doi:10.3390/nu18121951)
Supplement: Supplementary file 1 [file nutrients-18-01951-s001.zip › Supplementary Table S2 revised.pdf]

Supplementary Table S2. Sociodemographic, clinical treatment, and lifestyle characteristics according to UPF consumption categories

Variable	Total (*n*=425)	Low UPF (*n*=233)	High UPF (*n*=192)	*p*-value
**Marital status**				0.845
Single	67 (15.8)	36 (15.5)	31 (16.1)	
Married	358 (84.2)	197 (84.5)	161 (83.9)	
**Educational level**				0.822
Illiterate/primary school	254 (59.8)	138 (59.2)	116 (60.4)	
Secondary/high school	112 (26.4)	64 (27.5)	48 (25.0)	
University or higher	59 (13.9)	31 (13.3)	28 (14.6)	
**Income status**				0.788
Income higher than expenses	28 (6.6)	14 (6.0)	14 (7.3)	
Income equal to expenses	190 (44.7)	107 (45.9)	83 (43.2)	
Income lower than expenses	207 (48.7)	112 (48.1)	95 (49.5)	
**Diabetes treatment type**				0.716
Oral antidiabetic drugs	294 (69.2)	165 (70.8)	129 (67.2)	
Insulin	34 (8.0)	18 (7.7)	16 (8.3)	
Oral antidiabetic drugs + insulin	97 (22.8)	50 (21.5)	47 (24.5)	
**Hypoglycemia in the last 3 months**				0.800
No	352 (82.8)	192 (82.4)	160 (83.3)	
Yes	73 (17.2)	41 (17.6)	32 (16.7)	
**Hypertension**				0.113
No	230 (54.1)	118 (50.6)	112 (58.3)	
Yes	195 (45.9)	115 (49.4)	80 (41.7)	
**Dyslipidemia**				**0.010***
No	164 (38.6)	77 (33.0)	87 (45.3)	
Yes	261 (61.4)	156 (67.0)	105 (54.7)	
**Smoking status**				0.257
Current smoker	107 (25.2)	57 (24.5)	50 (26.0)	
Non-smoker	293 (68.9)	166 (71.2)	127 (66.1)	
Former smoker	25 (5.9)	10 (4.3)	15 (7.8)	
**Alcohol use**				0.465
No	418 (98.4)	228 (97.9)	190 (99.0)	
Yes	7 (1.6)	5 (2.1)	2 (1.0)	
**Regular exercise**				0.158
No	302 (71.1)	159 (68.2)	143 (74.5)	
Yes	123 (28.9)	74 (31.8)	49 (25.5)	
**Sleep quality**				0.161

Variable	Total (*n*=425)	Low UPF (*n*=233)	High UPF (*n*=192)	*p*-value
Good	126 (29.6)	77 (33.0)	49 (25.5)	
Moderate	204 (48.0)	110 (47.2)	94 (49.0)	
Poor	95 (22.4)	46 (19.7)	49 (25.5)	
**Sugar-sweetened beverage consumption**				**0.001***
Never/rarely	237 (55.8)	147 (63.1)	90 (46.9)	
1–3 times/month	101 (23.8)	54 (23.2)	47 (24.5)	
1–2 times/week	64 (15.1)	26 (11.2)	38 (19.8)	
>3 times/week	7 (1.6)	2 (0.9)	5 (2.6)	
Daily	16 (3.8)	4 (1.7)	12 (6.3)	
**Fast-food consumption**				0.096
Never/rarely	351 (82.6)	196 (84.1)	155 (80.7)	
1–3 times/month	38 (8.9)	23 (9.9)	15 (7.8)	
1–2 times/week	33 (7.8)	14 (6.0)	19 (9.9)	
>3 times/week	3 (0.7)	0 (0.0)	3 (1.6)	
**Packaged snack consumption**				**0.001***
Never/rarely	267 (62.8)	161 (69.1)	106 (55.2)	
1–3 times/month	102 (24.0)	55 (23.6)	47 (24.5)	
1–2 times/week	48 (11.3)	13 (5.6)	35 (18.2)	
>3 times/week	6 (1.4)	3 (1.3)	3 (1.6)	
Daily	2 (0.5)	1 (0.4)	1 (0.5)	

*Data are presented as n (%). Percentages are column percentages within UPF consumption categories. Categorical variables were compared using Pearson's chi-square test; Fisher's exact test was used for alcohol use because of sparse expected cell counts. *p<0.05. UPF, ultra-processed food.*
